# Around the Hospitals

**Published:** 1895-06-01

**Authors:** 


### AROUND THE HOSPITALS.

[NOTICE TO THE MANAGERS OF INSTITUTIONS.—Special space is now reserved for the insertion of notices of hospital meetings and festivals. The Editor requests that all notices may reach THE HOSPITAL Office, 428, Strand, at least one week before the date of each meeting.]

**Royal Surrey County Hospital, Guildford.**—At the annual meeting of the Royal Surrey County Hospital, Guildford, a most satisfactory financial condition was made apparent. The report showed a large increase in the general income, and a credit balance. A special appeal at a county meeting had resulted in nearly £1,500 being raised. A further sum of £1,700 was still needed for the building of a nurses' block, and the Hon. St. John Brodrick, M.P., announced that a gentleman had promised to give £500 towards it if a similar sum could be raised from other sources. The Bishop of Winchester, who presided at the meeting, started a subscription list with a donation of £25, and several other governors also contributed.

**Essex and Colchester General Hospital.**—Judging from the report of this hospital for the past year, there is much room for improvement in the management. The hospital is not a large one, receiving under 600 patients during the year; but, taking this into consideration, it must be admitted that £5 0s. 4½d. as an average cost per patient is a high one. This sum is partly accounted for by the long stay averaged by the patients, which is nearly forty days per head. The committee state they are giving the matter their earnest attention, and were the uniform system of accounts adopted their task would be an easier one, as they would then be able by comparison with other hospital accounts to detect in what direction over expenditure lay. The average cost of out-patients, too, comes out far too highly. Two-and-sixpence is now regarded a fair sum for a general hospital, but at Colchester 4s. 6¾d. is laid to the charge of each out-patient. We regret to note the omission of a comparative table of the income and expenditure for several years, which now usually finds its way into the yearly record of most hospitals, and is often instructive. The report is interesting, and the index is a useful feature in it. Several structural improvements have been effected throughout the building. During the past year the in-patients numbered 595 and the out-patients 2,071, both showing a decrease on the previous year's figures. The income amounted to £4,735, and the expenditure to £3,553 £657 was invested.

